# Student, instructor, and observer agreement regarding frequencies of scientific teaching practices using the Measurement Instrument for Scientific Teaching-Observable (MISTO)

**DOI:** 10.1186/s40594-018-0128-1

**Published:** 2018-08-16

**Authors:** Mary F. Durham, Jennifer K. Knight, Emily K. Bremers, Jameson D. DeFreece, Alex R. Paine, Brian A. Couch

**Affiliations:** 1 School of Biological Sciences, University of Nebraska, 204 Manter, Lincoln, NE 68588-0118 USA; 2 Department of Molecular, Cellular, and Developmental Biology, University of Colorado, Boulder, CO 80309 USA; 3 Biology Department, Doane University, Crete, NE 68333 USA

**Keywords:** Active Learning, Assessment, Inclusivity, Metacognition, Science process skills, Science practices, Scientific Teaching, Teaching practices, Undergraduate

## Abstract

**Background:**

The Scientific Teaching (ST) pedagogical framework encompasses many of the best practices recommended in the literature and highlighted in national reports. Understanding the growth and impact of ST requires instruments to accurately measure the extent to which practitioners implement ST in their courses. Researchers have typically relied on students, instructors, or observers to document course teaching practices, but it remains unclear whether and how these perspectives differ from each other. To address this issue, we modified our previously published instrument to generate the Measurement Instrument for Scientific Teaching-Observable (MISTO), which can be completed by students, instructors, and observers, and we investigated the degree of similarity between these three perspectives across 70 undergraduate science courses at seven different institutions in the USA.

**Results:**

We found that the full MISTO and Active Learning subcategory scores showed the highest correlations among the three perspectives, but the degree of correlation between perspectives varied for the other subcategories. Match scores between students and instructors were significantly higher than observer matches for the full MISTO and for the Active Learning, Inclusivity, and Responsiveness subcategories.

**Conclusions:**

We find that the level and type of agreement between perspectives varies across MISTO subcategories and that this variation likely stems from intrinsic differences in the course access and scoring decisions of the three perspectives. Building on this data, we recommend MISTO users consider their research goals, available resources, and potential artifacts that may arise when deciding which perspective best fits their needs in measuring classroom teaching practices.

**Electronic supplementary material:**

The online version of this article (10.1186/s40594-018-0128-1) contains supplementary material, which is available to authorized users.

## Background

Undergraduate science education is in the midst of broad-scale efforts to shift teaching and learning approaches from traditional lecture-style instruction to more active, evidence-based strategies that foster student success (American Association for the Advancement of Science (AAAS) 2015; Freeman et al. 2014; National Research Council (NRC) 2003, 2012; President’s Council of Advisors on Science and Technology (PCAST) 2012). Professional development programs play a major role in facilitating this change by training instructors in effective pedagogies and best practices. National-level programs have been established in several disciplines including the Geosciences (Manduca et al. 2010), Chemistry (Baker et al. 2014), Physics and Astronomy (Henderson 2008), and Biology (Ebert-May et al. 2015; Pfund et al. 2009; Wood and Handelsman 2004). The Scientific Teaching (ST) pedagogy encapsulates many of the best practices highlighted in these workshops. ST aims to engage students in the process of science and encourage instructors to use data to inform their instructional decisions. ST includes a wide range of research-based instructional strategies organized into three main pillars: Active Learning, Assessment, and Inclusivity (Handelsman et al. 2007).

In previous work, we developed a taxonomy of observable ST practices to identify and delineate the various pedagogical goals and instructional techniques of ST (Couch et al. 2015). Briefly, ST practices reflect a backward design approach to align learning objectives with group activities and formative assessments that foster student engagement (Frederick 1987; Prince 2004; Wiggins and McTighe 2005). ST enables the success of all students through the use of inclusive teaching practices, such as reducing unconscious biases and stereotype threats (Dasgupta and Greenwald 2001; Seymour 2000; Steele 1997; Tanner and Allen 2007; Uhlmann and Cohen 2005). ST also focuses on the development of science process skills in which students practice and communicate science (Bao et al. 2009; Coil et al. 2010; Goldey et al. 2012; Hanauer et al. 2006; Wei and Woodin 2011) and make connections between science and society (Chamany et al. 2008; Labov and Huddleston 2008; Pierret and Friedrichsen 2009; Sadler et al. 2004; Zeidler et al. 2005). Finally, ST prioritizes certain cognitive skills, such as higher-order thinking (Bloom et al. 1956), interdisciplinary reasoning (Bialek and Botstein 2004; Labov et al. 2010; Tra and Evans 2010), and metacognitive reflection (Ertmer and Newby 1996; Pintrich 2002; Schraw et al. 2006; Tanner 2012).

To better gauge the impacts of professional development and other transformation efforts on undergraduate science education, valid and reliable measurement instruments are needed to document the current state of undergraduate science classrooms, monitor how teaching changes over time, and determine what student outcomes result from any changes (Gess-Newsome et al. 2003; Smith et al. 2013; Wieman and Gilbert 2014). We recently published a survey called the Measurement Instrument for Scientific Teaching (MIST), designed to gauge the frequencies of ST practices in undergraduate science courses (Durham et al. 2017). This instrument provides estimates of the degree of implementation for the ST pedagogy overall and within each of eight ST subcategories: Active Learning Strategies, Learning Goal Use and Feedback, Inclusivity, Responsiveness to Students, Experimental Design and Communication, Data Analysis and Interpretation, Cognitive Skills, and Course and Self Reflection. MIST questions were designed with minimal jargon and worded in the third-person so that students, instructors, or observers can all potentially respond to each question given comparable exposure to the course.

Understanding differences between student, instructor, and observer perspectives is important because each of these three perspectives has potential benefits and limitations for measuring instructional practices. Students have the ability to report on how they experience a course, but they are commonly criticized for infusing personal biases in surveys. For example, instructor characteristics, including gender, age, and sense of humor, as well as external factors, such as the weather on the day of the survey, have been found to influence student responses on course evaluations (Becker and Watts 1999; Braga et al. 2014; Spooren et al. 2013). Instructors have more pedagogical expertise than students, but they may over-report their use of research-based instructional strategies, especially after participation in professional development programs or when their results are related to promotion and tenure decisions (Ebert-May et al. 2011; Wieman and Gilbert 2014). Finally, while observers may have less cause for subjective biases, observations require substantial logistical coordination efforts as well as significant time, training, and personnel resources. Furthermore, observers naturally focus on what they see in class and only score a small sample of class sessions for a given course, which may or may not be representative of the entire course (Lund et al. 2015; Stains et al. 2018). Previous studies comparing these different perspectives have typically used different instruments to capture each perspective, preventing a direct comparison of the same items and scales.

In light of these issues, we sought to investigate the degree of alignment between student, instructor, and observer perspectives when documenting course practices using MIST. Since observers are necessarily limited to a small sample of classes, we created a modified version of MIST, called MIST-Observable (MISTO), which includes only the ST practices and frequencies that can be detected in video samples from 1 week of class sessions. We measured (1) to what degree student mean, instructor, and observer MISTO scores correlate with each other across subcategories, (2) how closely these three perspectives estimate the amount of class time devoted to active learning, and (3) how closely the three perspectives match on individual items and whether agreement varies across subcategories. Understanding the relationships between these perspectives will help researchers, instructors, and administrators better interpret course measurement data and identify the perspective that aligns most closely with their goals.

## Methods

### Data collection

We collected survey data and video recordings from 70 courses at seven U.S. institutions (Table 1). We first used our professional networks and conference presentations to recruit site coordinators at seven institutions, and these coordinators then identified individual instructors at their own institutions who were interested in participating. We attempted to recruit instructors with a wide range of teaching styles from low to high ST implementation. Students and instructors completed MIST online outside of class near the end of the semester via Qualtrics survey software. Instructors were asked to offer their students a small amount of course credit to incentivize survey completion. Instructors were video recorded for 1 week of class sessions, which consisted of 2–3 separate class sessions and typically 150 min of class time. We included student data from 68 of these courses in our previous work (Durham et al. 2017).

**Table 1:** MISTO administration demographics

	Number	% of sample
Institutions	7	
Carnegie classification		
Highest research activity (R1)	5	56%
Higher research activity (R2)	2	33%
Undergraduate enrollment		
Medium (10,000–20,000)	1	14%
Large (20,000–30,000)	3	43%
Very large (> 30,000)	3	43%
Courses	70	
Discipline		
Biology	68	97%
Other STEM	2	3%
Enrollment		
Small (< 25 students)	13	19%
Medium (26–100 students)	16	23%
Large (> 100 students)	41	58%
Course level		
Lower division (100–200 level)	34	49%
Upper division (300–400 level)	36	51%
Instructors	58	
Academic position		
Adjunct/lecturer	2	3%
Contract-based lecturer	14	24%
Tenure-track lecturer	1	2%
Assistant professor	13	22%
Associate professor	9	16%
Professor	19	33%
Age		
30–39	16	28%
40–49	10	17%
50–59	18	31%
60–69	11	19%
70 or over	2	3%
Gender		
Female	24	41%
Male	34	59%
Ethnicity		
Underrepresented minority (URM)	3	5%
Non-URM	55	95%
Native language		
Non-English	5	9%
English	52	91%
Teaching experience		
First semester	3	5%
1–2 years	5	9%
3–5 years	11	19%
6–10 years	9	16%
11–15 years	8	14%
16–20 years	5	9%
Over 20 years	16	28%
Number of teaching training events (past 5 years)		
None	9	16%
1–2	18	31%
3–4	10	17%
5 or more	21	36%

### Development of MIST-Observable

To produce a version of MIST amenable to class observation, we first identified and removed 12 items referring to practices that occur outside of class time, such as out-of-class homework (Additional file 1). This led to the elimination of the Learning Goal subcategory because the associated practices generally took place outside of class time or through course documents. Observational studies generally use a small sample of class sessions to gauge teaching practices for a course; so to accommodate a typical sample size, we designed MISTO for use with 1 week of video recorded class sessions (Lund et al. 2015; Lund and Stains 2015). The original MIST contains items with response frequencies that could not be used by an observer based on a 1-week observation period (e.g., an observer could not say that something happened once per month). Thus, any implementation frequencies of less than once per week were removed from the response scales. This change applied to 27 out of the 36 MISTO items. We refer to the resulting survey containing a reduced item set and modified response scales as MIST-Observable (MISTO; Table 2). We note that the question prompts do not change between instruments but only the response scales are reduced to reflect a 1-week observation timeframe (Table 3).

**Table 2:** MISTO questions

Item	Cat.^1^	MISTO questions
Q1	ALS	Indicate the average percent of class time during which students were asked to answer questions, solve problems, or complete activities other than listening to a lecture
Q2	None	Learning goals were provided for
Q3	ALS	Students were asked to use a polling method to answer questions in the classroom approximately
Q4	ALS	Indicate the approximate percent of polling questions for which students were asked to discuss the question in pairs or small groups
Q5	ALS	Students were asked to complete in-class activities approximately
Q6	None	Indicate the approximate percent of in-class activities for which students were given some form of general or individualized feedback during class beyond simply providing correct or incorrect answers
Q7	None	Students were asked to work in groups of two or more for any portion of this course
Q8	ALS	Indicate the average percent of class time during which students were asked to work in groups of two or more
Q9	ALS	Students were asked to work in groups of two or more on in-class activities, discussions, assignments, or projects other than polling questions approximately
Q10	ALS	The instructor used a strategy, such as assigning roles, to promote the participation of each group member during in-class group activities
Q11	ALS	At least some students were asked to verbally share the results of any group work or group discussions with the whole class approximately
Q12	ALS	Students were asked to comment or make suggestions on each other’s work on class assignments, activities, or projects approximately
Q13	ALS	Students were encouraged to respond to classmates’ ideas during whole-class discussions
Q14	Inc	Examples or analogies used in this course included a diversity of people and cultures
Q15	Inc	Students were encouraged to consider the ideas and contributions of a diversity of researchers and other people involved in science
Q16	RtS	Students stated interests or asked questions related to the topic at hand during class
Q17	RtS	The instructor was generally aware of instances when a concept was not understood by the majority of students in the class prior to an exam
Q18	RtS	When it became clear that the class did not understand a concept, students were provided with follow-up discussion, activities, or resources
Q19	EDC	Students were asked to identify or formulate hypotheses or make predictions about the results of demonstrations, experiments, or examples approximately
Q20	EDC	Students were asked to critique scientific hypotheses or experimental strategies approximately
Q21	EDC	Students were asked to design experiments to answer scientific questions approximately
Q22	DAI	Students were asked to summarize, interpret, or analyze data using mathematical or computational procedures approximately
Q23	DAI	Students were asked to make graphs or tables approximately
Q24	DAI	Students were asked to analyze or interpret scientific data shown in graphs or tables approximately
Q25	DAI	Students were asked to use data to make decisions or defend scientific conclusions approximately
Q26	DAI	Students were asked to make or interpret models to summarize scientific processes approximately
Q27	EDC	Students were asked to interpret or critique scientific literature or media articles related to science approximately
Q28	EDC	Students were asked to communicate scientific ideas in formal written papers or oral presentations approximately
Q29	RtS	Students were provided with examples or explanations showing that course concepts are applicable to everyday human experiences or real-life applications approximately
Q30	None	Historical context was used to recognize why certain discoveries or advancements changed the way people viewed related scientific principles approximately
Q31	CS	Students were asked to interpret or represent concepts in non-written formats, such as pictures, diagrams, videos, simulations, role plays, graphs, mathematical models, etc.
Q32	CS	Students were asked to practice knowledge or skills from other Science, Technology, Engineering, and Math (STEM) subjects when answering questions or completing class activities
Q33	CS	Students engaged in higher level thought processes that required them to apply, analyze, incorporate, or evaluate their knowledge or skills rather than just memorizing facts or processes approximately
Q34	CS	Students were asked to participate in open-ended exercises, such as case-studies or questions in which multiple correct answers are possible
Q35	CSR	Students were provided with opportunities or suggestions to reflect on whether their study habits were effective for learning approximately
Q36	CSR	Students were provided with opportunities or suggestions to reflect on their problem-solving strategies approximately

^1^ MISTO subcategory abbreviations: *ALS* Active Learning Strategies, *Inc.* Inclusivity, *RtS* Responsiveness to Students, *EDC* Experimental Design and Communication, *DAI* Data Analysis and Interpretation, *CS* Cognitive Skills, *CSR* Course and Self Reflection

**Table 3:** Response scale conversion from MIST to MISTO^1^

Finite frequency style responses							
Example question	Students were asked to make graphs or tables approximately						
MIST response choices	Zero times	1–2 times during the semester	About 1 time per month	2–3 times per month	1–2 times per week	3–4 times per week	More than 4 times per week
MISTO response choices	Zero times	(Eliminated)	(Eliminated)	(Eliminated)	1–2 times per week	3–4 times per week	More than 4 times per week
General frequency style responses							
Example question	The instructor was generally aware of instances when a concept was not understood by the majority of students in the class prior to an exam						
MIST response choices	Not at all	Rarely	Less than half of the time	Half of the time	More than half of the time	Most of the time	Always
MISTO response choices	Not at all	(Eliminated)	Less than half of the time	Half of the time	More than half of the time	(Eliminated)	Always

^1^Yes/no, 0–100% slider bars, and Likert style agree-disagree scales did not change between MIST and MISTO

**Fig. 1:**
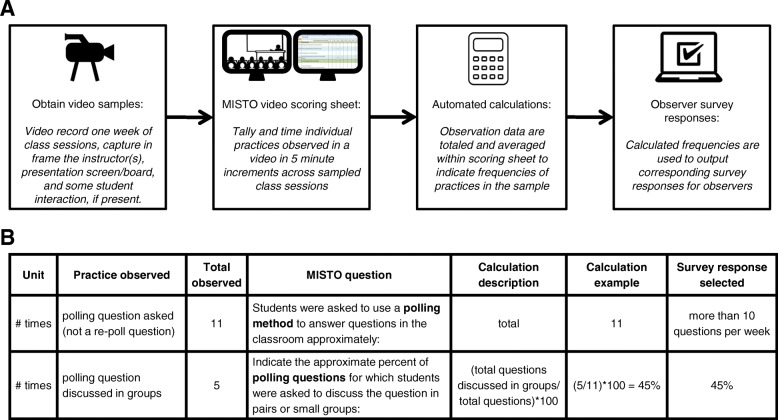
MISTO video observation process. **a** Diagrammatic representation of the observer scoring process. Observers score videos from class sessions using MISTO video scoring sheets. Embedded formulas in the workbook calculate observer survey responses and scores. **b** Hypothetical example of how teaching practices in a video sample are converted to observer survey responses. Here, polling questions are totaled and used to answer the corresponding survey question resulting in a survey response of “10 or more questions per week.” The number of polling questions discussed in groups is totaled and then used to calculate the percent of polling questions discussed in groups, which results in an observer survey response of 45%

To standardize observations, we created a MISTO video scoring workbook to record teaching practices and convert these counts to observer survey responses (Fig. 1). Observers use a scoring sheet to indicate the specific ST practices that occur in 5-minute intervals throughout class sessions. Observers record the number and duration of activities but do not record the quality or nature of the teaching practices. Because MISTO scores the number rather than the overall presence/absence of practices, fine granularity over time was not required. We found that the 5-minute timeframe partitioned the course video into manageable increments without overburdening the observer. An Excel file containing the observer video scoring workbook can be found in Additional file 2. The video scoring workbook file contains a separate scoring sheet for each class session along with descriptions of how to score each practice. Embedded formulas calculate the frequencies, durations, and proportions of teaching practices in the video sample, and these values are used to generate observer MISTO responses.

### MISTO scoring and match scores

Prior to analysis, we transformed the original student and instructor MIST survey responses to the new MISTO instrument by eliminating the non-observable items and converting any reported frequencies of less than once per week to zero. MISTO subcategory scores could then be calculated similarly for student, instructor, and observer data using the approaches previously described for MIST (Durham et al. 2017). Briefly, response categories were converted to ordinal values (e.g., “zero times” = 0, “1–2 times per week” = 1, “3–4 times per week” = 2, “more than 4 times per week” = 3), and each survey response (ordinal or continuous) was normalized to the maximum possible score for that survey item (e.g., a question with response values of 0, 1, 2, and 3 would be divided by 3). Scores from all questions included in each subcategory were averaged and normalized to a scale of 100 for each course. Thus, MISTO scores and subcategory scores could potentially range from 0 to 100. Low MISTO scores reflect less than weekly implementation of most practices, mid-range MISTO scores reflect weekly implementation of all practices or daily implementation of some practices, and high MISTO scores reflect daily implementation of all ST practices. We note that even very high ST users will not reach the top of the scale since it is not realistic to implement all the practices multiple times in every class.

We aimed to compare all three perspectives (students, instructors, and observers) with no a priori assumption of which perspective would serve as the reference point. Thus, we derived “match scores” to estimate how closely aligned responses were between two perspectives. Match scores for an item were calculated using the following equation:

$$ 1-\left(\left(|{\mathrm{score}}_1-{\mathrm{score}}_2|\right)/\mathrm{maximum}\ \mathrm{score}\right), $$

where score_1_ and score_2_ represent the scores assigned by each perspective. For students, each item score was the mean student response for a course. Match scores were then averaged for the full MISTO and each subcategory for each course. Match scores occur on a scale of 0–1 with a higher match indicating closer agreement between perspectives.

### MISTO observer training and agreement

Once the video scoring rubric was formalized, we developed a training procedure to achieve acceptable agreement between observers. Initially, two observers co-coded 1 week of videos from a “training set” of eight courses. These two observers monitored their agreement and discussed any disagreements to consensus. Two additional observers separately scored the eight courses from the training set, progressing from more guided scoring to more independent scoring across the eight courses. The observers monitored agreement with the consensus scores for these videos and discussed any disagreements to consensus.

Following training, all observers were tested for acceptable agreement. One observer first coded 1 week of videos from five new courses. Next, each of the other three raters coded three of these courses, and all three of the observers achieved an average match score above 0.75, which we considered sufficient for independent scoring. Again, any disagreements were discussed to consensus.

The videos from the remaining courses in this study were each scored by one of the four observers. After all initial course observations were completed, two of the observers co-coded a set of ten courses to check whether acceptable agreement had been maintained, achieving an average match score of 0.94 across the ten courses.

### Statistical analyses

All statistical analyses were carried out using R (Core Team 2016). We used the cor.test function to investigate Pearson’s product moment correlations between variables. We tested for differences in match scores between perspectives using ANOVA with pair-wise post hoc Tukey’s tests using the TukeyHSD function. Effect sizes, reflected by Hedge’s *g*, were estimated using the cohen.d and hedges.correction functions in the effsize package (Torchiano 2015). We tested for relationships between instructor and course characteristics with match scores using *t* tests and ANOVAs.

### Human subjects research approval

This project was classified as exempt from Institutional Review Board review at UNL (project ID 15016), CU (project ID 15-0297), and all other participating institutions.

## Results

### Correlation of MISTO scores between perspectives

**Fig. 2:**
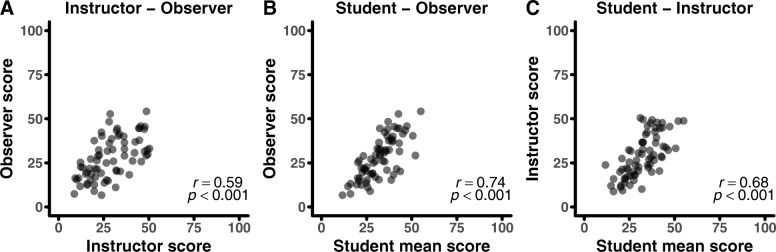
Full MISTO score correlations between perspectives. Correlations between **a** instructor and observer scores, **b** student mean and observer scores, and **c**. student mean and instructor scores. Dots correspond to MISTO scores for each course; *n* = 70 total courses. MISTO scores can range from 0 to 100

**Fig. 3:**
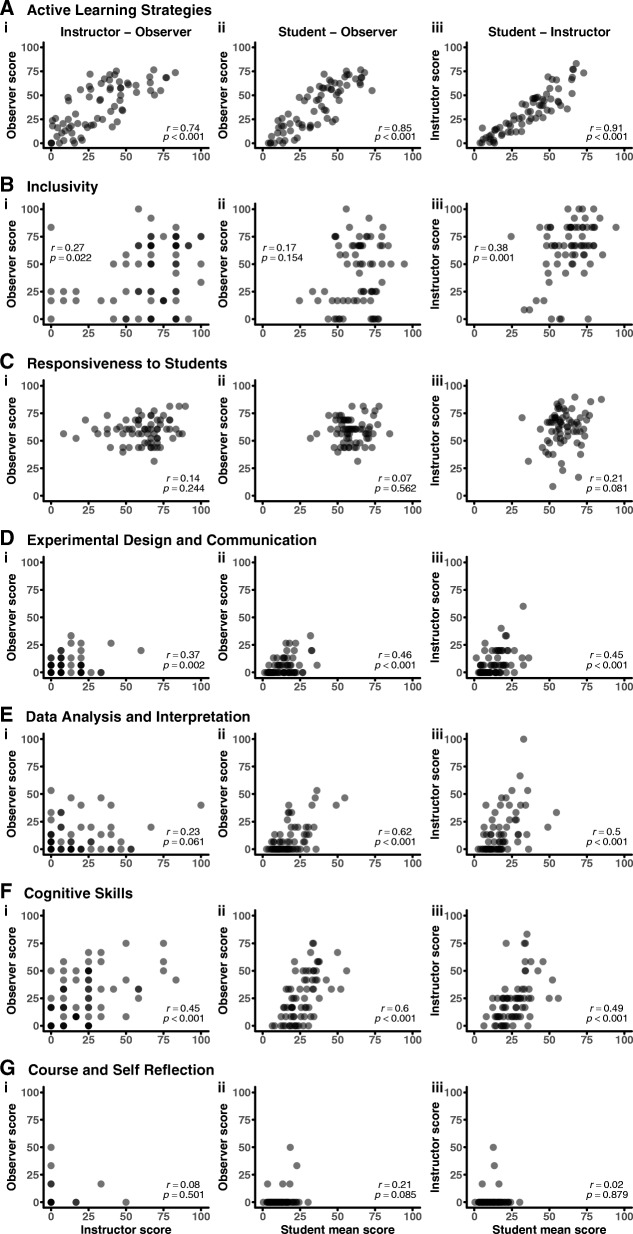
MISTO subcategory score correlations between perspectives. Rows represent each of seven MISTO subcategories: **a** Active Learning Strategies, **b** Inclusivity, **c** Responsiveness to Students, **d** Experimental Design and Communication, **e** Data Analysis and Interpretation, **f** Cognitive Skills, **g** Course and Self Reflection. Columns represent correlations between (i) instructor and observer scores, (ii) student mean and observer scores, and (iii) student mean and instructor scores. Dots correspond to MISTO subcategory scores for each course; *n* = 70 total courses. MISTO subcategory scores can range from 0 to 100

MISTO scores showed varying degrees of correlation between perspectives and those correlation levels varied among the full and subcategory scales (Figs. 2 and 3). Based on established guidelines for defining correlation levels (Jackson 2013), full MISTO scores showed moderate to strong correlations (*r* = 0.59–0.74, *p* < 0.001; Fig. 2). The Active Learning subcategory scores showed strong correlations between all perspectives (*r* > 0.7, *p* < 0.001), with the highest correlations occurring between students and instructors (Fig. 3a). The remaining subcategories showed moderate (*r* = 0.3–0.7) to low (*r* < 0.3) correlations between perspectives, and these levels varied by pairings (Fig. 3). The Responsiveness and Reflection categories showed no significant correlations between perspectives.

### Correlation of active learning estimations between perspectives

**Fig. 4:**
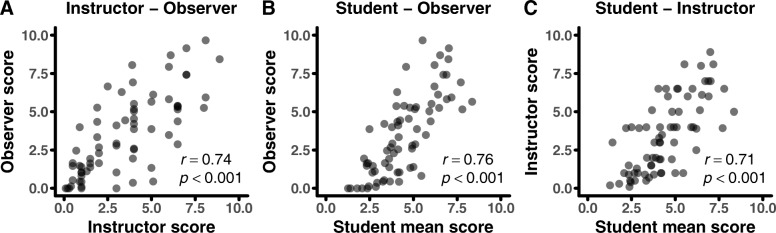
Correlations in estimates of percent active learning between perspectives. Correlations between **a** instructor and observer responses, **b** student mean and observer responses, and **c** student mean and instructor response for proportion active learning item. Dots correspond to item response for each course; *n* = 70 total courses

Researchers have also used more targeted measures of the percent of class time in which active learning takes place as a proxy for the degree of transformed teaching (Owens et al. 2017, 2018; Smith et al. 2013). Thus, we also calculated correlations for a single item asking respondents to “indicate the average percent of class time during which students were asked to answer questions, solve problems, or complete activities other than listening to a lecture” and found strong correlations (*r* > 0.7) between all perspectives (Fig. 4).

### Match between perspectives is often high but varies among MISTO subcategories

**Fig. 5:**
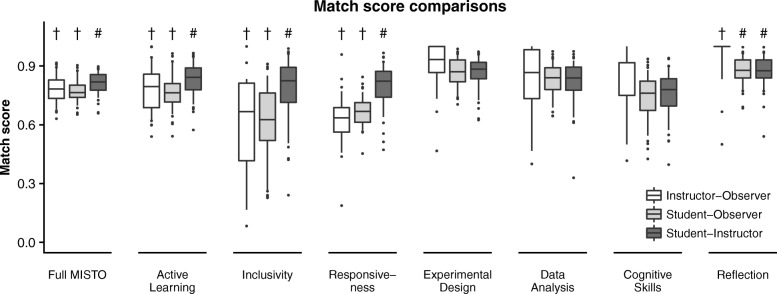
Full MISTO and MISTO subcategory match scores comparing perspectives. Average item match scores among MISTO items on the full MISTO and on each MISTO subcategory. Box plots show the distributions of average MISTO and subcategory match scores for each course between two perspectives: instructor–observer comparisons (white boxes), student–observer comparisons (light gray boxes), and student–instructor comparisons (dark gray boxes); *n* = 70 courses. Central bars represent median match scores, boxes reflect interquartile range, whiskers reflect the 5th and 95th percentile range, and dots represent data points outside this range. Different symbols represent significant differences between perspectives within the full MISTO or within each MISTO subcategory, as determined by ANOVA with pair-wise post hoc Tukey tests

To better understand agreement between perspectives, we also used the “match scores” described in the methods to determine how closely the perspectives scored each item relative to its own scale. All three perspectives showed relatively high matches, with most pair-wise comparisons matching above 0.75. The relative match level varied between perspective pairs for several MISTO subcategories (Fig. 5 and Additional file 3). Student–instructor match scores were significantly higher than the student–observer and instructor–observer matches for the full MISTO, Active Learning, Inclusivity, and Responsiveness subcategories (all *p* < 0.01). The instructor–observer match was significantly higher than either student match for Reflection (*p* < 0.001), and no significant differences were observed between perspectives for the Experimental Design, Data Analysis, and Cognitive Skills subcategories.

We also investigated the effect of several course and instructor characteristics on match scores (Additional file 4) and identified no significant differences in match scores based on the instructor’s gender, age, number of years teaching, or number of pedagogical training events recently attended (*p* = 0.07–0.89). We also found no significant influence of class size or course level on agreement (*p* = 0.21–0.87).

### Similarity among perspectives for an example course

**Fig. 6:**
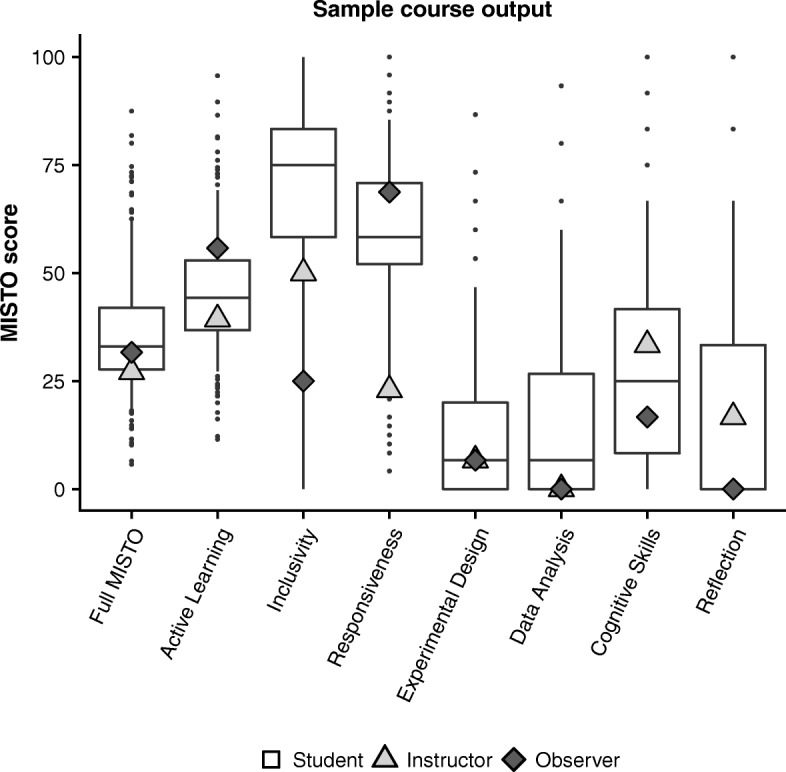
Sample MISTO course output. Example output of student, instructor, and observer MISTO scores for an example course. Light gray triangles represent the instructor scores, and the dark gray diamonds represent the observer score. Box plots represent student responses; *n* = 310 students. Central bars represent median scores, boxes reflect interquartile range, whiskers reflect the 5th and 95th percentile range, and dots represent data points outside this range

We have included the MISTO score output from an example course to illustrate the range of variation seen in student responses and how instructors and observers compare to the distribution of student scores (Fig. 6). In this example course, the instructor and observer scores fell within the inner quartile range of student scores for the full MISTO and five of the MISTO subcategories. The instructor indicated a lower score than the lower quartile of student responses in the Inclusivity and Responsiveness subcategories. The observer also indicated an Inclusivity score lower than the lower student quartile; however, the observer score was higher than the student upper quartile for Active Learning (Fig. 6).

## Discussion

Building on our previously developed MIST survey, we created an observer-compatible version called MISTO to capture frequencies of ST practices in undergraduate science courses, along with a video scoring workbook to facilitate observation. We used MISTO to measure ST in videos of class sessions from 70 courses, and we compared results among students, instructors, and observers to investigate the degree of similarity in MISTO and subcategory scores between these perspectives. To our knowledge, this study represents the first instance in which these three perspectives were compared using an equivalent instrument.

### Agreement between perspectives varies among subcategories

We found the full MISTO and subcategory scores fell into three general types of agreement between perspectives: (1) high correlation–high match, (2) moderate correlation–high match, and (3) low correlation–low match.

The full MISTO and Active Learning subcategory scores showed high correlations and high match scores between perspectives (Figs. 2, 3, and 5). This was particularly striking for student–instructor agreement in the Active Learning subcategory, where there was a nearly one-to-one correlation (*r* = 0.91). Previous studies have raised potential concerns regarding the shortcomings and limitations of sampling teaching practices from each of the perspectives measured in this study (Braga et al. 2014; Ebert-May et al. 2011; Lund et al. 2015; Spooren et al. 2013). Despite these criticisms, the high correlations and high match scores across perspectives for the full MISTO and Active Learning suggest that students, instructors, and observers can produce comparable scores.

The Experimental Design, Data Analysis, and Cognitive Skills subcategories all showed moderate correlations and high matches (Figs. 3 and 5). The moderate correlations indicate that each perspective produces somewhat different scores, but the high matches suggest consistency in responses to individual items relative to the response scales. This apparent discrepancy can be partly explained by the low overall implementation levels in the courses sampled for these subcategories. In other words, the perspectives generally agreed that a set of practices occurred infrequently, but there was variation in quantifying the precise level of implementation. Taken together, the correlations and matches of these three subcategories indicate that each perspective may have the capacity to score the occurrence of these practices similarly, but the practices did not occur frequently enough to fully support this claim. This same rationale may also apply to the Reflection subcategory, which represents a more extreme case since it only occurred in a few of the 70 courses.

Finally, the Inclusivity and Responsiveness subcategories showed low correlations and low matches (Figs. 3 and 5). The low agreement between perspectives for these categories can be attributed, at least in part, to the agree-disagree response scales used for these questions. However, we note that despite the inherent subjectivity of these types of scales, the student–instructor match scores were above 0.75 and significantly higher than either of the observer match scores. This suggests that the observers might be missing nuanced features of these practices or misinterpreting classroom culture.

### Student–instructor comparisons showed the highest agreement

The student–instructor comparisons showed the highest match scores and strongest correlations for three subcategories (Active Learning, Inclusivity, and Responsiveness) as well as the highest match scores for the full MISTO (Figs. 3 and 5). While individual students may have misestimated ST practice frequencies, the central tendencies in their responses fell into closer agreement with the instructor scores. This provides evidence that, as a whole, students were generally aware of and attuned to what was happening in the course and that their perceptions aligned with instructors. Furthermore, the lower matches in the observer comparisons suggest that students and instructors may be using slightly different criteria to answer these items or that certain practices occurred outside the purview of the observer. For example, students and instructors may incorporate student–instructor rapport when answering Responsiveness items, whereas observers followed strict criteria for scoring instances of instructor feedback. Additionally, students and instructors may have been including practices that occurred outside of class time (e.g., online feedback or discussion boards), while observers were limited to the in-class portion of a course.

### Benefits and challenges of each perspective

Overall, our results highlight the nuanced nature of alignment between student, instructor, and observer responses for the full MISTO and subcategory scores. Given these results, we recommend that MISTO users consider their research goals and the resources available when deciding which perspective (s) to use as a measure of ST implementation.

Students provide the benefit of larger sample sizes from which to elicit a measure of central tendency to estimate ST levels. Students represent a universally available resource for all courses, and collecting student responses can help mitigate potential conflicts with instructor motivations. For example, if an instructor wished to collect documentation of their teaching practices for promotion and tenure purposes, data from their students would likely be seen as more credible than self-reported data. As the ultimate target of educational programs, student perspectives also carry a certain primacy of importance. If students report a particular set of practices (e.g., inclusivity) at a lower frequency than instructors or observers, one would want to further investigate the reasons behind this phenomenon. On the negative side, student data tends to be noisy on the individual student level, and collecting data from students requires some additional coordination and potentially institutional review board (IRB) considerations.

Instructors represent the most practical and accessible option for gathering data, especially under conditions of limited resources or when data collection spans multiple institutions. Since instructor surveys can be collected without any student involvement, the study coordination and IRB approval processes are substantially more streamlined. This may be a major motivation driving the use of instructor self-reports to measure teaching practices in many studies (Borrego et al. 2013; Dancy and Henderson 2010; Eagan et al. 2014). Instructors also potentially have deeper insights into design features and course content than either of the other two perspectives. Professional development program facilitators may wish to use the survey as a formative experience to help instructors reflect on their teaching (Gormally et al. 2014). Conversely, researchers aiming to evaluate professional development programs may want to avoid using instructor responses as their only data source because instructors could inflate their scores if they feel pressure to convey the success of the program (Ebert-May et al. 2011).

From the research perspective, observers represent the most standardized data collection approach. In particular, observers can be trained to score courses according to explicit criteria, and their reliability can be gauged through co-coding with other observers. In this regard, they minimize many of the potential biases and item interpretation issues intrinsic to the other perspectives. Conversely, it must be acknowledged that course observations require significant resources that may exceed the means of many investigators and departments. Obtaining approval, collecting class videos, training observers, and scoring videos all become increasingly cost and time prohibitive as the number of courses and institutions in a study grows. Furthermore, observers are also affected by their potential inability to observe out-of-class practices, interpret course norms, or understand course content.

While the prospect of collecting data on teaching practices from any perspective may seem daunting, we intend for the data presented here to enable investigators to make more informed decisions based on the intrinsic benefits and empirical differences between perspectives. We hope that this type of reflection will help researchers and departments more effectively leverage their available resources to achieve their desired goals. For example, a department wishing to document ST practices for institutional reporting may choose to administer MIST to students because it approximates data from the other perspectives, requires fewer resources than observations, and avoids potential suspicions regarding instructor self-reports. Furthermore, we hope that the supporting materials we have provided here and in our previous publications will help alleviate the logistical barriers to using MIST and MISTO (Couch et al. 2015; Durham et al. 2017).

### Other considerations

Research documenting course practices faces practical and intrinsic challenges that warrant consideration. This study used a sample of 70, primarily biology, courses at seven US institutions, so the broader generalizability of the conclusions remains to be determined. In comparing across perspectives, we had to make several necessary concessions based on observers only being able to view 1 week of class and only viewing in-class events. In particular, the conversion of MIST responses for students and instructors to MISTO response scales caused a general lowering of scores because low implementation frequencies were reduced to zero. Furthermore, eliminating questions that principally occur outside of class resulted in MISTO collecting less information than MIST. Thus, although adjustments were made to enable observation-based comparisons, we propose that the original MIST version provides a more thorough representation if users only wish to collect data from students or instructors.

We also note an unavoidable difference between MIST and MISTO: in MIST, students and instructors were asked to reflect cumulatively on the whole semester, whereas in MISTO, observers focused only on a particular week of class sessions. Several other studies have used a 1-week sample (Lund et al. 2015; Lund and Stains 2015), and the high correlations for the Active Learning Strategies between students/instructors (who answered based on experiencing nearly a whole semester) and observers (who answered based on 1 week of class) suggests that the degree of activity during a 1-week timeframe was fairly representative of the broader semester. However, the inherent differences in the level of detail and accuracy of these reflections represent a limitation that should be considered when interpreting the results. Although MISTO was designed and used here for 1-week samples, adjustments can be made to the survey response scales and scoring sheets to support shorter or longer observation periods.

In developing MIST, we recognized that some items and subcategories were inherently more susceptible to variation in interpretation. For example, the Inclusivity questions are measured on an agree-disagree scale, which incorporates some personal interpretation, whereas the Active Learning subcategory relies on numerical counts of discrete events. In general, the different levels of agreement across subcategories could be explained by the relative objectivity of the items. Thus, when considering the range of current and future instruments available, one would predict that instruments focusing on objective and recognizable practices (e.g., clicker questions) will have greater potential for agreement between perspectives than those with more subjective and nuanced practices (e.g., scoring whether instructors incorporated scenarios reflecting diverse perspectives). We also note that practices associated with the Experimental Design, Data Analysis, and Reflection subcategories were implemented quite infrequently in our sample. These practices are likely to be important features of science curriculum, so their low levels of implementation warrant further research for the broader field.

## Conclusions

As transformation efforts in undergraduate science education continue, measurements of teaching practices are needed to gauge the status of the field, track how teaching changes over time, and determine the impact of specific strategies (Freeman et al. 2014; Gess-Newsome et al. 2003; Smith et al. 2013; Wieman and Gilbert 2014). To support this effort, the MIST and MISTO instruments were designed to measure frequencies of teaching practices associated with the ST pedagogy, which encompasses many of the best practices recommended by science education research (Couch et al. 2015; Durham et al. 2017; Handelsman et al. 2007). By developing MISTO, we laid a foundation for a comparison of classroom practices from three different perspectives (i.e., students, instructors, and observers) using a single set of items. Our results indicate that all three perspectives produce relatively similar estimations of the full MISTO and Active Learning subcategory but exhibit different levels of agreement for the other subcategories. We found that student–instructor data were often more closely aligned than either perspective was to observers. More broadly, our work supports claims that survey and observation instruments designed using objective and easily interpreted questions can elicit relatively accurate estimations of teaching practices and agreement between perspectives, especially when conducted in low-stakes environments (Wieman and Gilbert 2014), whereas agreement between perspectives may be more difficult to achieve for more complex practices.

### How to use MISTO

We have included the MISTO video scoring workbook (Additional file 2). While the workbook contains specific criteria for scoring each practice, we have found that many practices occur simultaneously and learning to keep track of these many aspects requires practice. Depending on incoming expertise in observations or ST, we estimate about 8–12 h of watching and scoring class sessions could adequately prepare observers for independent scoring. Once observers have completed the scoring workbook for a course, they can use the scoring template to calculate MISTO and MISTO subcategory scores for each perspective measured (Additional file 5). While we recommend the full MIST survey for students and instructors, we have included a Qualtrics file for cases where users wish to administer MISTO online to students or instructors (Additional file 6).

### Use and availability of MIST instruments

The suite of MIST instruments includes the full MIST, which is ideal for collecting student and instructor data, the MIST-Short, which is a shortened version of the survey that can be used in conjunction with other measures such as student learning or self-efficacy surveys, and MISTO, which is designed for observations and comparisons among perspectives. MIST and MIST-Short can be found in our previous publication (Durham et al. 2017); MISTO and the video scoring workbook can be found in the Additional files.

### Human subjects research approval

This project was classified as exempt from Institutional Review Board review at UNL (project ID 15016), CU (project ID 15-0297), and all other participating institutions.

## Additional files

Additional file 1: MIST items removed from MISTO. This file lists all the MIST survey questions that were removed from MIST in creating MISTO, generally because the associated ST practices were not observable or were inconsistently observable in video recordings of classroom sessions. (DOCX 14 kb)

Additional file 2: MISTO video scoring workbook. This file contains a multi-sheet Excel workbook where observers record teaching practices on up to three scoring sheets. Those records are then translated into observer MISTO survey responses and their corresponding scores for data analysis (see Fig. 1). (XLSX 360 kb)

Additional file 3: Summary of match score comparisons between perspectives. This file contains a table listing statistical analyses of perspectives pairs. Omnibus ANOVA results are shown on the left and pairwise Tukey HSD results are shown on the right. Significant differences in pairs are bolded. (DOCX 16 kb)

Additional file 4: Effects of course and instructor characteristics on match scores. Match pair indicates the perspectives being compared: IO is instructor–observer, SO is student–observer, and SI is student–instructor. (DOCX 16 kb)

Additional file 5: MISTO scoring template. After obtaining MISTO responses either through the online survey or the MISTO video scoring workbook, this Excel template can be used to calculate MISTO and MISTO subcategory scores for each perspective measured. Note: This template is designed for use with the MISTO question set (not the full MIST question set). (XLSX 2499 kb)

Additional file 6: MISTO Qualtrics file. This qsf file contains the MISTO survey, which can be administered to students or instructors using the online Qualtrics platform. This version of the survey should only be used when asking students or instructors to reflect on a one week sample of class sessions or when comparing these perspectives to observers. For other purposes, we recommend using the original MIST survey qsf file, which is available in the supplement of the original publication (Durham et al. 2017). (QSF 134 kb)

## Abbreviations

DBER: Discipline-based education research
MIST: Measurement Instrument for Scientific Teaching
MISTO: Measurement Instrument for Scientific Teaching-Observable
ST: Scientific Teaching
STEM: Science, technology, engineering, and mathematics
