# Supplementary material for: Student, instructor, and observer agreement regarding frequencies of scientific teaching practices using the Measurement Instrument for Scientific Teaching-Observable (MISTO)
Source: Int J STEM Educ. 2018 Aug 16;5(1):31. doi: 10.1186/s40594-018-0128-1 (PMC6310438; doi:10.1186/s40594-018-0128-1)
Supplement: Supplementary file 1 — MIST items removed from MISTO. This file lists all the MIST survey questions that were removed from MIST in creating MISTO, generally because the associated ST practices were not observable or were inconsistently observable in video recordings of classroom sessions. (DOCX 14 kb) [file 40594_2018_128_MOESM1_ESM.docx]

| **Additional File 1.** The following MIST^1^ items were removed in MISTO | | | |
| --- | --- | --- | --- |
| Item | Cat.^2^ | MIST questions |  |
| MIST Q4 | LGF | Indicate the approximate percent of **polling questions** that overlapped with the **learning goals** provided by the instructor: |  |
| MIST Q7 | LGF | Indicate the approximate percent of **in-class activities** that overlapped with the **learning goals** provided by the instructor: |  |
| MIST Q9 | ALS | Students were asked to complete **out-of-class assignments** approximately: |  |
| MIST Q10 | LGF | Indicate the approximate percent of **out-of-class assignments** that overlapped with the **learning goals** provided by the instructor: |  |
| MIST Q11 | LGF | Indicate the approximate percent of **out-of-class assignments** for which students were given some form of general or individualized **feedback** beyond simply providing correct or incorrect answers: |  |
| MIST Q12 | none | Students were asked to complete **major exams or term projects,** including final exams approximately: |  |
| MIST Q13 | LGF | Indicate the approximate percent of questions or components on **major exams or term projects** that overlapped with the **learning goals** provided by the instructor: |  |
| MIST Q14 | LGF | Indicate the approximate percent of questions or components on **major exams or term projects** for which students were given some form of general or individualized **feedback** beyond simply providing correct or incorrect answers: |  |
| MIST Q18 | ALS | Students were asked or encouraged to **work in groups** of two or more on **out-of-class** activities, assignments, or projects approximately: |  |
| MIST Q26 | Inc | The instructor was sensitive to socially controversial issues. |  |
| MIST Q27 | CSR | Students were asked to provide formal or informal feedback on course activities and content prior to the end of the semester evaluation. |  |
| MIST Q28 | CSR | Student feedback on course activities and content was used to make adjustments to the course within the semester: |  |
| ^1^ See Durham et al., 2017 for details on the MIST instrument.  ^2^ MIST subcategory abbreviations: ALS: Active Learning Strategies, LGF: Learning Goal Use and Feedback, Inc: Inclusivity, RtS: Responsiveness to Students, EDC: Experimental Design and Communication, DAI: Data Analysis and Interpretation, CS: Cognitive Skills, CSR: Course and Self Reflection. Note: LGF removed from MISTO. | | |  |
