# Supplementary material for: Student, instructor, and observer agreement regarding frequencies of scientific teaching practices using the Measurement Instrument for Scientific Teaching-Observable (MISTO)
Source: Int J STEM Educ. 2018 Aug 16;5(1):31. doi: 10.1186/s40594-018-0128-1 (PMC6310438; doi:10.1186/s40594-018-0128-1)
Supplement: Supplementary file 3 — Summary of match score comparisons between perspectives. This file contains a table listing statistical analyses of perspectives pairs. Omnibus ANOVA results are shown on the left and pairwise Tukey HSD results are shown on the right. Significant differences in pairs are bolded. (DOCX 16 kb) [file 40594_2018_128_MOESM3_ESM.docx]

**Additional file 3: Summary of match score comparisons between perspectives.** This file contains a table listing statistical analyses of perspectives pairs. Omnibus ANOVA results are shown on the left and pairwise Tukey HSD results are shown on the right. Significant differences are bolded.

|  | **ANOVA** | | ***Post hoc* Tukey's HSD** | | | |
| --- | --- | --- | --- | --- | --- | --- |
| **Subcategory** | *F*_(2, 207)_ | *p* | pairs | *T* | *p*-value | Hedges *g* |
| Full MISTO | 10.69 | **<0.0001** | SO-IO | -1.25 | 0.425 | 0.203 |
|  |  |  | SI-IO | 3.23 | **0.004** | -0.468 |
|  |  |  | SI-SO | 4.48 | **<0.0001** | -0.814 |
| Active Learning | 8.565 | **0.0003** | SO-IO | -0.95 | 0.609 | 0.150 |
|  |  |  | SI-IO | 3.01 | **0.008** | -0.419 |
|  |  |  | SI-SO | 3.96 | **0.0003** | -0.769 |
| Inclusivity | 19.18 | **<0.0001** | SO-IO | -0.14 | 0.989 | 0.022 |
|  |  |  | SI-IO | 5.29 | **<0.0001** | -0.714 |
|  |  |  | SI-SO | 5.43 | **<0.0001** | -1.153 |
| Responsiveness | 58.02 | **<0.0001** | SO-IO | 2.25 | 0.065 | -0.387 |
|  |  |  | SI-IO | 10.25 | **<0.0001** | -1.443 |
|  |  |  | SI-SO | 8.00 | **<0.0001** | -1.275 |
| Experimental Design | 3.47 | **0.03** | SO-IO | -2.28 | 0.061 | 0.362 |
|  |  |  | SI-IO | -2.29 | 0.059 | 0.300 |
|  |  |  | SI-SO | -0.01 | 0.999 | 0.003 |
| Data Analysis | 0.22 | 0.803 | not applicable | | | |
| Cognitive Skills | 1.34 | 0.263 | not applicable | | | |
| Reflection | 30.77 | **<0.0001** | SO-IO | -6.66 | **<0.0001** | 1.077 |
|  |  |  | SI-IO | -6.92 | **<0.0001** | 0.934 |
|  |  |  | SI-SO | -0.25 | 0.965 | 0.046 |
