# Supplementary material for: Student, instructor, and observer agreement regarding frequencies of scientific teaching practices using the Measurement Instrument for Scientific Teaching-Observable (MISTO)
Source: Int J STEM Educ. 2018 Aug 16;5(1):31. doi: 10.1186/s40594-018-0128-1 (PMC6310438; doi:10.1186/s40594-018-0128-1)
Supplement: Supplementary file 4 — Effects of course and instructor characteristics on match scores. Match pair indicates the perspectives being compared: IO is instructor–observer, SO is student–observer, and SI is student–instructor. (DOCX 16 kb) [file 40594_2018_128_MOESM4_ESM.docx]

**Additional File 4.** Effects of course and instructor characteristics on match scores. Match pair indicates the perspectives being compared: IO is instructor-observer, SO is student-observer, and SI is student-instructor.

| ***t-*test results** | | | | | | | | | | | | |
| --- | --- | --- | --- | --- | --- | --- | --- | --- | --- | --- | --- | --- |
| Variable | match pair | *μ*_female_ | | *μ*_male_ | | *df*_M_ | | *t* | | *p* | | *R* |
| Instructor gender | IO | 0.774 | | 0.789 | | 62.3 | | -1.0082 | | 0.3173 | | 0.127 |
|  | SO | 0.763 | | 0.777 | | 64.9 | | -1.1922 | | 0.2375 | | 0.146 |
|  | SI | 0.805 | | 0.820 | | 42.1 | | -1.0480 | | 0.3006 | | 0.159 |
|  | |  |  | |  | |  | |  | |  | |
| **ANOVA results** | |  |  | |  | |  | |  | |  |  |
| Variable | match pair | *df*_M_ | *df*_R_ | | F | | *p* | | *ω* | |  |  |
| Instructor age | IO | 1 | 67 | | 2.9550 | | 0.0902 | | 0.166 | |  |  |
|  | SO | 1 | 67 | | 3.4440 | | 0.0679 | | 0.185 | |  |  |
|  | SI | 1 | 67 | | 0.0200 | | 0.8870 | | 0.120 | |  |  |
| Class size | IO | 1 | 68 | | 0.1390 | | 0.7110 | | 0.112 | |  |  |
|  | SO | 1 | 68 | | 0.0280 | | 0.8690 | | 0.119 | |  |  |
|  | SI | 1 | 68 | | 0.2350 | | 0.6300 | | 0.105 | |  |  |
| Course level | IO | 1 | 68 | | 0.0640 | | 0.8020 | | 0.116 | |  |  |
|  | SO | 1 | 68 | | 0.3780 | | 0.5410 | | 0.095 | |  |  |
|  | SI | 1 | 68 | | 1.6190 | | 0.2080 | | 0.094 | |  |  |
| Years teaching | IO | 1 | 67 | | 0.0300 | | 0.8630 | | 0.119 | |  |  |
|  | SO | 1 | 67 | | 0.8370 | | 0.3630 | | 0.049 | |  |  |
|  | SI | 1 | 67 | | 0.0320 | | 0.8590 | | 0.119 | |  |  |
| Number of training events | IO | 1 | 68 | | 0.0510 | | 0.8230 | | 0.117 | |  |  |
|  | SO | 1 | 68 | | 1.4130 | | 0.2390 | | 0.077 | |  |  |
|  | SI | 1 | 68 | | 0.0460 | | 0.8300 | | 0.117 | |  |  |
